# Metal–Organic-Framework- and MXene-Based Taste Sensors and Glucose Detection

**DOI:** 10.3390/s21217423

**Published:** 2021-11-08

**Authors:** Ha Huu Do, Jin Hyuk Cho, Sang Mok Han, Sang Hyun Ahn, Soo Young Kim

**Affiliations:** 1 School of Chemical Engineering and Materials Science, Chung-Ang University, Dongjak-gu, Seoul 06974, Korea; hadohuu1311@gmail.com; 2 Department of Materials Science and Engineering, Institute of Green Manufacturing Technology, Korea University, Seongbuk-gu, Seoul 02841, Korea; jhyukcho1993@gmail.com; 3 Korea Institute of Geoscience and Mineral Resources, Yuseong-gu, Pohang 37559, Korea

**Keywords:** metal–organic frameworks, MXenes, taste sensors, electrochemical sensing

## Abstract

Taste sensors can identify various tastes, including saltiness, bitterness, sweetness, sourness, and umami, and have been useful in the food and beverage industry. Metal–organic frameworks (MOFs) and MXenes have recently received considerable attention for the fabrication of high-performance biosensors owing to their large surface area, high ion transfer ability, adjustable chemical structure. Notably, MOFs with large surface areas, tunable chemical structures, and high stability have been explored in various applications, whereas MXenes with good conductivity, excellent ion-transport characteristics, and ease of modification have exhibited great potential in biochemical sensing. This review first outlines the importance of taste sensors, their operation mechanism, and measuring methods in sensing utilization. Then, recent studies focusing on MOFs and MXenes for the detection of different tastes are discussed. Finally, future directions for biomimetic tongues based on MOFs and MXenes are discussed.

## 1. Introduction

Tastes can be recognized by the human tongue via tasters. However, exposing human subjects to unknown and potentially dangerous products poses a health risk. Another way to evaluate taste is to employ a biochemical taste sensor called an electronic tongue. Before electronic tongues were first reported, Toko et al. published a patent for a taster prepared from a lipid-based membrane in 1989 [1]. Subsequently, the term “taster” was coined by Di Natale and co-workers. Winquit et al. prepared and innovated a hybrid electrochemical sensor [2], and Wang’s group functionalized a glassy carbon electrode (GCE) using three different materials as electrochemical sensors for the detection of rice wines [3]. Taste consists of five primary tastes: sweetness, bitterness, saltiness, sourness, and umami. Each of these tastes can be identified by the presence of a specific analyte. Notably, sour tastes are characterized by acidic protons; meanwhile, NaCl is an analyte for the determination of salty tastes. Molecules such as sucrose and glucose give rise to sweet tastes; bitterness is attributed to molecules such as caffeine, whereas monosodium glutamate is an example of a compound that creates an umami taste (Table 1). Generally, taste sensors have been utilized in the pharmaceutical, medical, environmental, food, and beverage fields by virtue of the importance in assessing products in these fields. Commercial systems of taste sensors have been based on electrochemical methods using a lipid membrane. Chemical components of lipid membrane influence the charge density and hydrophilicity of membrane surface, which is different for each taste sensor. For instance, in terms of the sourness sensor, the membrane surface needs to be created with good hydrophilicity and zero-charge to identify in acidic solution. The membrane surface requires a positive charge and hydrophobicity to accelerate hydrophobic interaction with the analyte to recognize the bitterness. The working mechanism of the kind of sensor is based on the physicochemical interaction between lipid membrane and analyte, causing the altering of the membrane potential [2]. The current commercial bioelectronic tongues are high-performing, but their drawback is their high cost [4].In the past few years, MOFs and MXenes have emerged as competitive candidates for the preparation of highly selective, ultra-stable, and low-priced sensors for particular applications [5,6,7,8,9].

MOFs have shown great potential in different applications such as photo-electrochemical catalysis, medicine, gas storage, and sensing by virtue of their excellent characteristics, including ultra-large surface area, adjustable structural components, and good thermal and chemical durability [10,11,12,13,14,15,16,17,18,19,20,21]. For example, DUT-60 is well-known for its high porosity and surface area of up to 7839 m^2^/g [22,23]. PCN-224 (Co) displayed outstanding durability under various acid concentrations [24], and Banerjee et al. introduced a Li-based MOF with ultrahigh thermal stability (610 °C) [25]. Moreover, MOFs are constructed from metal ions and organic ligands that can interact with other substances to generate response signals. Various interactions, including hydrogen bonds, electron donating/accepting interactions, and hydrophobic associations, can be initiated, whereas open metal sites can help stimulate chemical and electrostatic interactions. Furthermore, an outstanding property of MOFs is their tunable pore size, which can be adjusted by controlling the size of the precursors. This characteristic is favorable for the detection of target substances, which have various sizes. Therefore, MOFs have been used to prepare efficient and durable sensors to detect biomolecules, small-molecule gases, solvents, heavy-metal cations, and pesticides, making them indispensable in medical and environmental analysis. The potential of MOFs for electrochemical, fluorescent, and electromechanical sensing have been reported in many studies. For example, Ni-MOF-74, having unsaturated Ni sites in a durable porous material, was applied for the detection of glucose [26]. This strategy was employed by Xiao (2017), who used Ni-MIL-77 nanobelts as an electrochemical sensor for measuring the glucose content in a real blood sample [27]. Zn(II)-MOF has also been modified by amino groups to create an optical sensor for the determination of the Cr^6+^ cation and 2,4,6-trinitrophenol [28]. The effectiveness of amino-functionalized MOFs was demonstrated by Lu et al. [29], who prepared NH_2_-MIL-53(Al) and applied it as an optical sensor for the determination of ClO^–^ anions. In addition, Cu-based MOFs are good electromechanical sensors. In one study, Cu_3_(BTC)_2_ was deposited on an Au template as an electromechanical sensor for the detection of volatile organic compounds [29]. Although MOFs can be directly employed, their poor conductivity and low selectivity are limitations for researchers attempting to develop novel materials for sensing applications. Thus, numerous studies have focused on developing various MOF hybrid structures in order to enhance the sensing performance of MOFs.

MXenes, a young member of the two-dimensional (2D) materials group, are formed from the exfoliation of MAX-phase layers. MAX phases have the formula M_n + 1_AX_n_ (where M and A are transition metals Sc, Y, Ti, Ta, Mn, Mo, etc.; X is C or N; and *n* = 1, 2, 3, 4) are well known [5]. Owing to the diversity of structural components, numerous MAX phases have been introduced. As a result, many materials of the MXenes group with the general formula M_n+1_XT_x_ have been successfully prepared [30,31,32,33], where Tx is a polar species such as fluorine, hydroxyl, or oxygen. MXenes are prepared via three processes: etching, delamination, and intercalation. Etching is conducted by mixing the MAX phase with hydrofluoric acid (HF) in ambient conditions. The concentration of HF plays a critical role in controlling the morphology and structural component of MXenes, which give rise to their different chemical and physical properties. In particular, Alhabeb and co-workers investigated the change in morphology of T_3_C_2_T_x_ by varying the proportion of HF [34]. In addition, MXenes could be prepared from non-MAX phases, which was first reported by Meshkian and co-workers [35]. The author immersed Mo_2_Ga_2_C layers in HF to remove Ga atoms for the formation Mo_2_C. Zhou et al. utilized HF to etch Zr_3_Al_3_C_5_ precursor for fabrication 2D Zr_3_C_2_T_z_, which exhibited higher structural durability than Ti_3_C_2_T [36]. Overall, MXenes have good hydrophilicity, which facilitates their modification to enhance their properties [37,38,39]. In addition, they display an array of outstanding features, such as tunable surface characteristics, good mechanical properties, and high electrical conductivity [40,41]. Therefore, the past decade has witnessed the rapid development of MXenes in many different areas, including the catalysis [42,43,44], sensors [45,46,47], and supercapacitors field [48,49,50]. The collective results demonstrate the potential of MOFs and MXenes as excellent templates for taste sensors in particular and sensing applications in general.

This review reports the latest progress in MOF- and MXene-based sensors for taste assessment and glucose detection. We also briefly state the significance of taste sensors and explain their working principles and determining methods in sensing utilization. Subsequently, recent works related to MOFs and MXenes for detecting different tastes are discussed, followed by future directions for biomimetic tongues based on MOFs and MXenes.

## 2. Fundamentals of Taste Sensor

**Figure 1:**
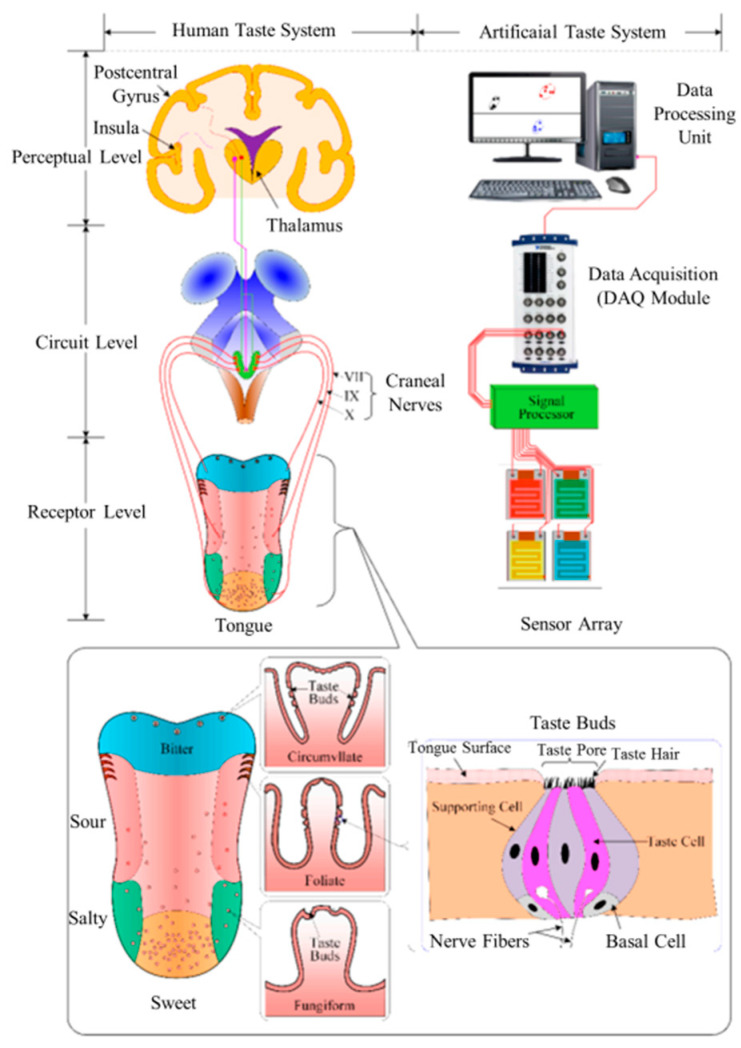
Taste sensing system of a human tongue compared to that of an electronic sensor. Reproduced with permission from Ref. [51].

The sweetness of cake and sour taste of vinegar are known to originate from the tongue; however, the mechanism of taste formation is a complex process. Figure 1 illustrates the formation of taste in the human body alongside an artificial taste system. Generally, the detection of taste includes three steps. In the biological tongue, the taste buds, called taste receptors, identify substances in our mouths; subsequently, dozens of taste cells generate and transmit electrical signals to the taste center of our brains (gustatory cortex) via axons. Our gustatory cortex provides information about taste. In terms of artificial taste systems, membranes, transducers, and computers function as the taste buds, nerves, and the thalamus, respectively, of the human body. Moreover, the multiple areas of the human tongue exhibit varying sensitivities. To illustrate, sweet tastes can be identified with the highest sensitivity on the side of the tongue, whereas bitter tastes are felt on the back of the tongue.

**Figure 2:**
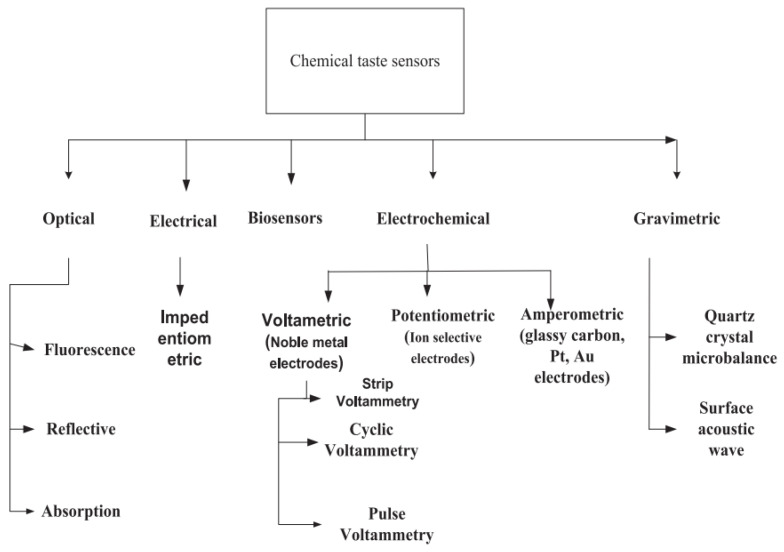
Various types of taste sensors have been adopted in electronic sensing. Reproduced with permission from Ref. [7].

To date, many types of taste sensor exist, which are classified based on their determination method. Notably, five types have been adopted in artificial taste systems: optical, electrical, biosensing, electrochemical, and gravimetric (Figure 2).

## 3. MOFs for Taste Sensing

### 3.1. MOFs as Taste Sensors

**Figure 3:**
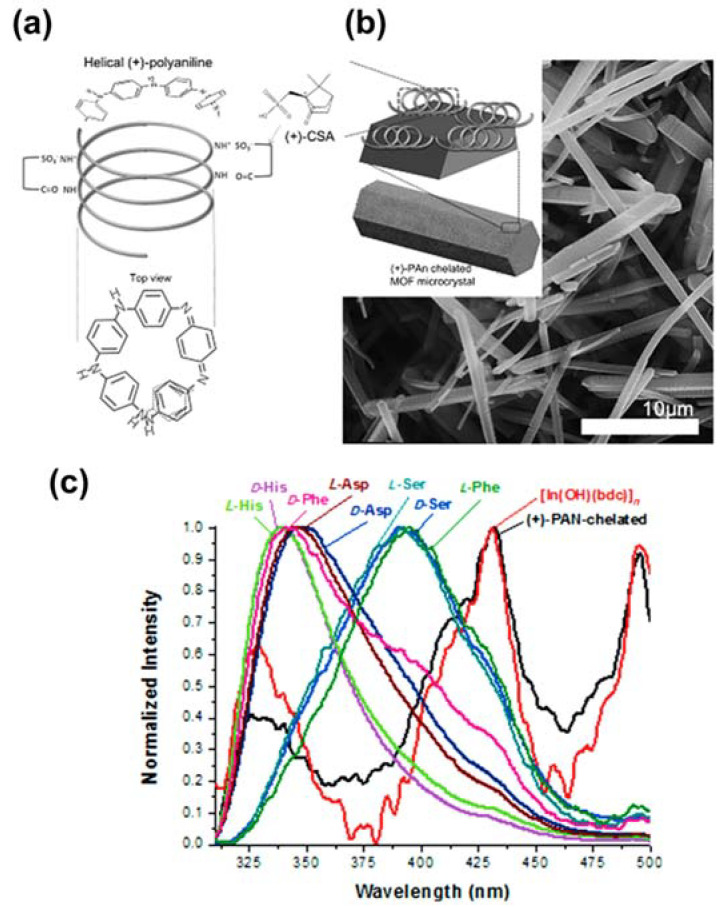
(**a**) Helical (+)-PAN structure, (**b**) scanning electron microscopy (SEM) image of (+)-PAN-chelated [In(OH)bdc]_n_, and (**c**) PL responses of [In(OH)bdc]_n_ and (+)-PAN-chelated [In(OH)bdc]_n_. Reproduced with permission from Ref. [56].

Photoluminescent (PL) MOFs have been used in various applications, such as light-emitting diodes (LEDs), sensors, and heavy-metal detection devices by reason of their metal node and linker components, which give rise to PL signals [52,53,54]. MOFs were applied to taste applications for the first time in 2013 by Lee and co-authors, who used three photoluminescent MOFs as biochemical taste sensors: MOF-76, [In(OH)(bdc)], and [Ca_3_(btc)_2_(DMF)_2_(H_2_O)]·3H_2_O [55]. [In(OH)(bdc)] and MOF-76 have relatively small a pore size for inclusion of analytes (e.g., sucrose). Notably, the pore sizes are 6.6 Å × 6.6 Å and 11.9 Å × 23.3 Å for MOF-76 and [In(OH)(bdc)], respectively. While sizes of sucrose is 9.0–9.8 Å. For this reason, the authors used poly(acrylic acid) to coat on the surface of [In(OH)(bdc)] to improve discrimination of chemical stimuli. Their study revealed that -COOH groups in PAA play a vital role in interaction with the analyte, leading to changes in the PL response, whereas the analyte formed hydrogen bonds with water molecules, which was integrated into the MOF-76 structure. Moreover, In^3+^ acts as an active site, reacting with reagents to obtain responses, whereas [Ca_3_(btc)_2_(DMF)_2_(H_2_O)]·3H_2_O plays a vital role in enhancing the taste detection performance. Later, Lee et al. used [In(OH)(bdc)] as a template to graft polyaniline as a biological tongue, as shown in Figure 3a,b [56]. The authors utilized L-phenylalanine (L-Phe) as a tastant for bitterness, whereas D-phenylalanine (D-Phe) was the analyte for sweetness. L-Phe and D-Phe can both interact with (+)-PAN, altering the PL peaks (Figure 3c). Furthermore, the authors did not observe any signals in the S 2p X-ray photoelectron spectra when D-Phe and L-Phe interacted with (+)-PAN-chelated [In(OH)(bdc)]. These results confirmed the generation of (+)-PAN on the MOF structure; Phe was absorbed on (+)-PAN-chelated [In(OH)(bdc)]. Moreover, various types of polymers containing polyvinylpyrrolidone, poly (acrylic acid), and sodium alginate have been used to functionalize the surface of [In(OH)(bdc)] to create bonds with tastants such as NaCl, caffeine, and sucrose, allowing them to operate as biosensors for taste detection.

### 3.2. MOF-Based Electrochemical Sensors for Glucose Detection

Sweetness is one of five tastes that play a vital role in stimulating humans to ingest nutrients. Accordingly, several studies have investigated the use of glucose as an analyte for sweetness-sensing applications [57,58,59].

#### 3.2.1. Pristine MOFs

MOFs with open metal centers have been employed for catalytic and sensing applications owing to their interaction ability [60]. For example, Lopa and co-workers chose CPO-27(Ni) as an active material for glucose detection using an electrochemical technique [26]. They found that this MOF has a high sensitivity of 40.95 µA/mM. Their results also suggested that Ni^2+^ is converted into Ni^3+^, which oxidizes molecular glucose. The effectiveness of Ni-based MOFs for glucose sensing has also been proven in the research conducted by Xiao et al., who found that Ni-MIL-77 yielded a sensitivity of 1.542 μA mM^−1^ cm^−2^ in basic media [27]. In addition, this research group also proposed a mechanism of glucose oxidation that was similar to that of CPO-27(Ni). Another example of Ni-based MOFs for glucose detection was reported by Qiao and co-workers [61]. They employed a π-conjugated 2,3,6,7,10,11-hexahydroxytriphenylene linker to prepare a conductive MOF using the solvothermal method. This MOF could detect glucose with a good sensitivity of 21.744 mA mM^−1^ cm^−2^. Although Ni-based MOFs have demonstrated great potential for glucose determination, their low electrical conductivity weakens their performance for electrochemical applications. Therefore, many different strategies have been devised to improve their conductivity and thus their glucose detection activity, such as by doping with noble metals and employing supported carbon materials. For instance, Peng et al. deposited silver nanoparticles on CPO-27(Ni) as an active material for non-enzymatic glucose sensing [62]. This material was coated on the surface of a GCE as an electrochemical sensor, which displayed an outstanding glucose detection performance, yielding a sensitivity of 1.29 mA mM^−1^ cm^−2^ and a limit of detection (LOD) of 4.7 µM. In another study, Chen et al. adopted gold nanograins to modify Ni-BTC for enhancing glucose determination performance [63]. Au/Ni-BTC was also coated on a GCE as a working electrode for the electro-oxidation of glucose. As expected, this electrode displayed good activity, with a linear range of 0.005–7.4 mM and a response sensitivity of 1.4471 mA mM^−1^ cm^−2^. Another performance-enhancing strategy is to use carbon materials such as reduced graphene oxide (rGO) or carbon nanotubes (CNTs) to accelerate carrier transport for the electrochemical process. An example of this strategy was described by Zhang et al. [4]. They fabricated a Ni-MOF/CNTs composite using a hydrothermal process and then deposited it on a GCE for use an anode for glucose oxidation. As expected, this sensor displayed a negligible electron-transfer resistance compared with that of Ni-MOF and CNTs. Therefore, it had a high glucose detection performance with a LOD of 0.82 µM. These experiments reveal that Au, Ag nanograins, and conductive carbon materials are beneficial for improving the sensing performance of MOFs.

**Figure 4:**
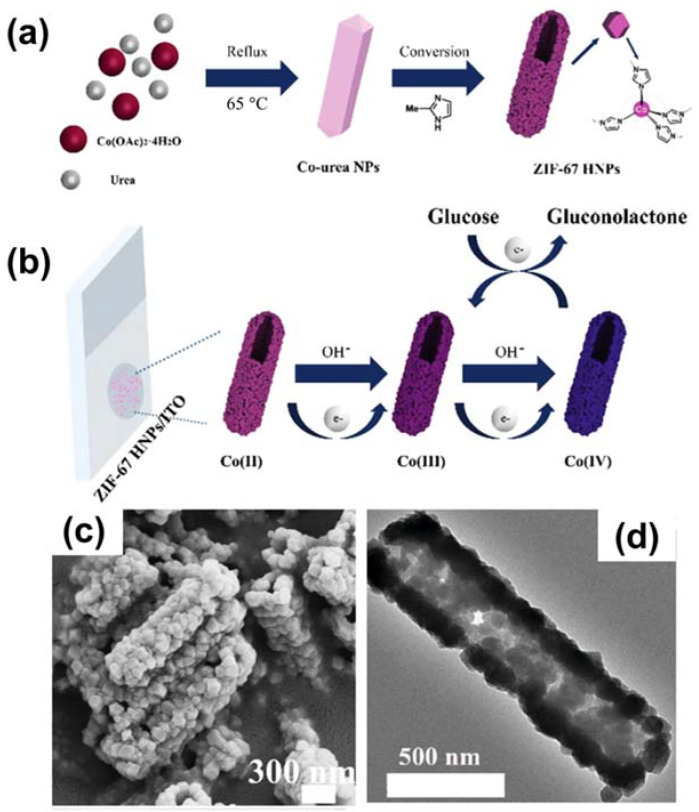
(**a**) Schematic illustrating the preparation of ZIF‒67 HNPs. (**b**) Mechanism of glucose oxidation on ZIF‒67 HNPs/ITO. (**c**) SEM and (**d**) transmission electron microscopy (TEM) images of ZIF‒67 HNPs. Reproduced with permission from Ref. [64].

Similar to Ni-based MOFs, Co-based MOFs have also been applied in glucose determination. In particular, ZIF-67 is a good example of a Co-MOF used for the electro-oxidation of glucose. Chen et al. fabricated ZIF-67 hollow structures (ZIF-67 HNPs), which they used to functionalize an indium tin oxide substrate to obtain a biosensor for glucose (Figure 4a,b) [64]. The morphological architecture of the MOF was investigated using scanning electron microscopy (SEM) and transmission electron microscopy (TEM), as shown in Figure 4c,d. This sensor showed good catalytic properties for the electro-oxidation of glucose in basic media, with a LOD of 0.96 µM.

**Figure 5:**
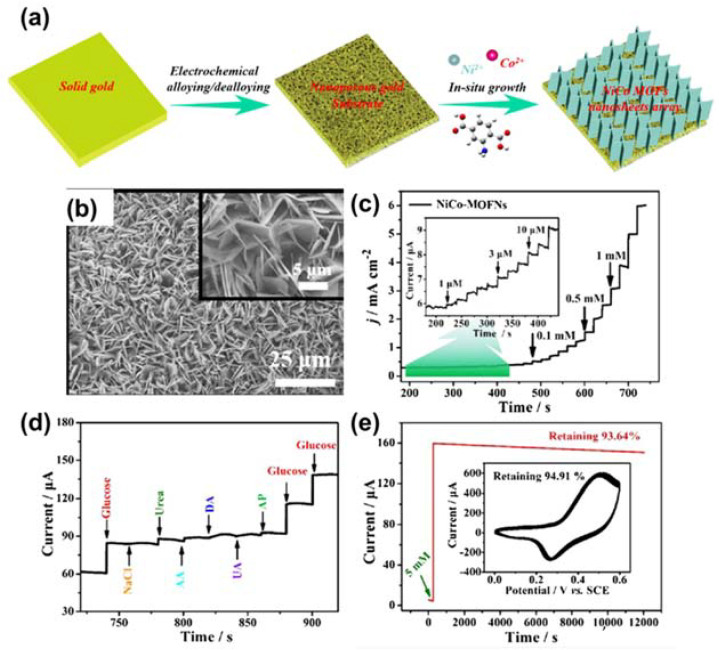
(**a**) Schematic illustrating the preparation of a NiCo‒MOF nanoflake. (**b**) SEM image of NiCo‒MOF nanoflake. (**c**) Current–time response of the NiCo‒MOF nanoflake with continuous addition of glucose in 0.1 M NaOH at 0.5 V vs. SCE. (**d**) Current–time response for the continuous addition of interfering species (0.1 mM) and glucose (1 mM) at 0.5 V. (**e**) Durability of the NiCo‒MOF nanoflake. Reproduced with permission from Ref. [71].

Various approaches have also been shown to improve the catalytic properties of Co-MOF for glucose electro-oxidation. For example, Meng and co-workers inserted silver nanograins into a ZIF-67 framework to improve its electrocatalytic properties [65]. This material displayed a low LOD of 0.66 µM and remarkable durability. They attributed this result to the accelerated charge-carrier transport of ZIF-67 by silver nanograins. In addition, Yuniasari et al. utilized graphene to improve electron transport in a type of Co-MOF [66]. As expected, this strategy produced exciting results, including a small detection limit of 5.39 µM and outstanding durability. Wei et al. presented a potential approach for the growth of cost-effective, flexible devices for glucose-sensing applications [67]. The author used Co-MOF to functionalize carbon cloth (CC)/paper as a three-dimensional (3D) electrode for glucose oxidation. This approach enhanced the electrochemical surface area compared to the GCE, leading to enhanced catalytic properties. As anticipated, this sensor displayed a low LOD of 150 µM. The effectiveness of CC was also demonstrated in a publication by Xu et al. [68]. The authors grew a Ni/Co-based MOF on CC as a flexible sensor for glucose using a simple hydrothermal technique. The sensor was effective in a wide linear range of 0.3–2312 µM. In addition, they used the sensor to determine glucose in beverages and obtained a high yield. Another bimetallic Ni/Co-based MOF was applied for glucose identification by Li and co-authors [69]. They varied the shape of the Ni/Co-MOF by adjusting the content of the precursor and obtained three different forms: flower, rod, film, and sheet-like. Film form yielded a good catalytic performance with a response sensitivity of 2.8 mA mM^−1^ cm^−2^. In addition to CC, nickel foam (NF) has also been utilized as a 3D template for the growth of MOF material as a working electrode in electrochemical reactions. For example, Li and co-workers deposited a Co-MOF on NF using the facile hydrothermal process to obtain a flexible sensor for glucose determination [70]. This strategy allowed for the exposure of active centers in the Co-MOF, improving the sensor performance; a sensitivity of 10,886 mA mM^−1^ cm^−2^ was recorded for glucose detection in alkaline solution. Li et al. coated a NiCo-MOF on a nanoporous gold template (Figure 5a), which operated as an enzyme-free biosensor for glucose diagnostics [71]. The nanoflake-shape of the NiCo-MOF was verified by SEM, as shown in Figure 5b. Figure 5c exhibits the current–time response of the NiCo-MOF nanoflake with continuous addition of glucose in 0.1 M NaOH. The data indicated that the sensor could detect glucose in a wide linear range of 1–8000 µM. Moreover, the selectivity of this sensor was evaluated by injecting NaCl, urea (Ure), L-ascorbic (AA), dopamine hydrochloride (DA), uric acid (UA), and acetaminophen (AP) in a glucose-containing medium (Figure 5d). Figure 5e reveals the outstanding durability of the sensor after 200 cycles and 12,000 s. Furthermore, the authors proposed a mechanism of glucose oxidation that involved the conversion of Ni^2+^ and Co^2+^ into Ni^3+^ and Co^4+^ moieties, respectively. These moieties oxidized glucose into gluconolactone. This strategy harnessed the effect of combining Ni and Co ions with a porous substrate. Another synergistic strategy was proposed by Zou et al., who prepared an ultrathin Co/Ni-MOF layer as an active material for glucose diagnostics [72]. Their results revealed that a Co/Ni ratio of 2:1 was optimal, leading to a low LOD, good durability, and outstanding selectivity. Notably, this sensor exhibited a response sensitivity of 2.0867 mA mM^−1^ cm^−2^ and a LOD of 0.047 µM. These merits were attributed to the efficient charge transport from Ni to Co in the MOFs.

**Figure 6:**
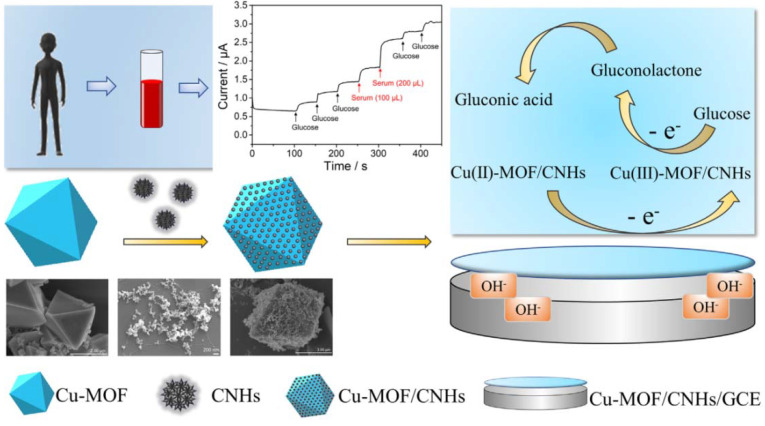
Schematic illustrating the synthesis of a Cu/MOF/CNHs-functionalized GCE and its utilization in glucose determination. Reproduced with permission from Ref. [76].

Cu- and Fe-based MOFs have also been applied for glucose diagnostics. For instance, Zheng et al. synthesized Cu-MOF on a carbon paste template (Cu-MOF/CPE) as an efficient anode for glucose recognition in basic solution [73]. This approach benefitted from the large surface area and high porosity of the MOF material, as well as the good conductivity of CPE. As a result, this device possessed a low LOD of 0.11 µmol/L, a linear range of 5–3910 µmol/L, and remarkable durability beyond 4000 s, determined using the chronoamperometric technique. This work implied that the CPE supports electron transport better than the GCE. For example, Sun et al. used an identical Cu-MOF coated on a GCE as a working electrode for the oxidation of glucose [74]. This sensor exhibited a LOD of 0.01 µmol/L and a linear range of 0.06–5 mM. To improve the performance of the Cu-MOF, several approaches were introduced by various research groups. Case in point: Liu and co-workers incorporated exfoliated graphene (EG) and Co-MOF to enhance catalytic activities for glucose oxidation [75]. This material demonstrated outstanding catalytic activity for glucose oxidation in the range 1.0–3330 µM and a LOD of 0.58 µM. The work also indicated that Co open-metal sites act as active catalytic moieties for glucose identification. Another example was reported by Zheng and co-workers, who used carbon nanohorns (CNHs) to accelerate the catalytic activity of a Cu-MOF for glucose diagnostics (see in Figure 6) [76]. The CNHs, with their large number of active centers and outstanding conductivity, significantly enhanced the performance of Cu-MOF for glucose recognition. In particular, this material displayed a linear range of 0.25–1200 µM with a LOD of 0.078 µM. The effectiveness of CNTs has been demonstrated in a publication by Wu et al. [77]. The authors functionalized a GCE with a Co-MOF and CNT, which acted as an efficient biosensor for glucose determination. Notably, this anode exhibited excellent catalytic properties in the range 0.5–11,840 µM and a small LOD of 0.4 µM. The use of CNTs imparts beneficial features such as abundant active moieties and high conductivity to the electrode material. Fe-based MOFs have also been applied in electrochemical glucose sensing; 2D Fe-BTC is one example of a Fe-based MOF used for this purpose. Yuan et al. synthesized Fe-BTC nanolayers under atmospheric conditions for the application of glucose diagnostics [78]. This MOF exhibited an outstanding catalytic activity for glucose electro-oxidation in the range 0.04–20 µM and a small LOD of 0.039 µM. This study also revealed that the performance of 2D-Fe-BTC is superior to that of 3D Fe-based MOF for glucose identification. For instance, Liu et al. synthesized a Fe-MIL-88NH_2_ nanocrystal as an active material for glucose recognition [79]. Their findings indicated that the synthesized nanocrystal had a linear range of 2.0–300 µM and LOD of 0.48 M. Another study that focused on a Fe-based MOF was presented by Dong and co-workers [80]. They showed that MIL-53(Fe) has good catalytic activities in the range 0.04–20 µM and a small LOD of 0.039 µM for glucose diagnostics.

#### 3.2.2. MOF-Derived Metal Compounds

**Figure 7:**
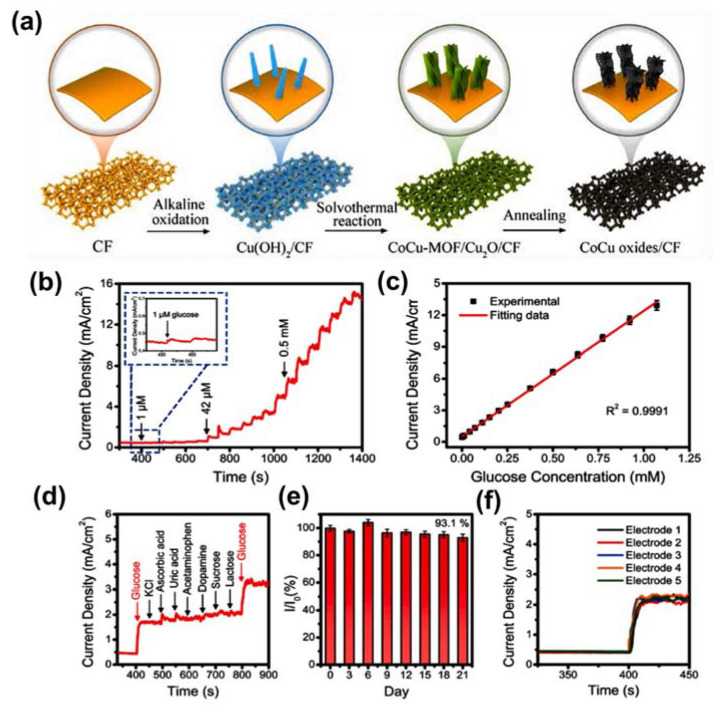
(**a**) Graphic illustration of the preparation of CoCu oxides/CF. (**b**) Amperometric curve for CoCu oxides/CF with continuous addition of glucose in 0.1 M NaOH at 0.5 V. (**c**) Corresponding calibration plot of current density vs. various glucose contents. (**d**) Amperometric curve of CoCu oxides/CF with continuous addition of 0.1 mM glucose, 0.02 mM K^+^, 0.02 mM AA, 0.02 mM UA, 0.02 mM AP, 0.02 mM DA, 0.02 mM sucrose, and 0.02 mM lactose. (**e**) Durability of CoCu oxides/CF sensor over 21 days. (**f**) Current–time curves for five sensors of CoCu oxides/CF for glucose detection. Reproduced with permission from Ref. [85].

In addition to using MOFs as active materials for glucose-sensing applications, many researchers have converted MOFs into metal compounds such as metal/carbon composites, metal oxides, and metal phosphides for glucose detection. For example, Zhang et al. fabricated Ni nanograins embedded in nickel foam (NF) as an electrochemical sensor for glucose diagnostics [81]. The authors deposited the Ni-MOF on NF before initiating the pyrolysis under argon flow to produce the Ni@C nanolayer. This electrode exhibited an amazing glucose sensing ability with a very low LOD of 50 nM and a linear range of 0.15–1480 µM. In addition to metal-based materials, metal oxides synthesized from MOFs have also been applied in glucose sensing. For example, Luo et al. utilized a Cu-based MOF to create CuO using a pyrolysis process [82]. This catalyst was coated on GCE, which operates as an anode for glucose oxidation, and exhibited outstanding activity with a low LOD of 0.15 µM and a high sensitivity of 1806.1 µA cm^−2^ mM^−1^. Muthurasu et al. deposited ZIF-67 on a CuO nanowire (NW) prior to initiating the oxidation to convert ZIF-67 into Co_3_O_4_. This strategy harnessed the effect of using a binary-metal-oxide system to yield high conductivity. As a result, glucose could be identified in a linear range of 0.5–100 µM and a low LOD of 0.23 µM. Vilian et al. improved the activity of Co_3_O_4_ for glucose detection by combining it with rGO [83]. This approach accelerated the adsorption of glucose on Co_3_O_4_/rGO, and its enhanced conductivity led to a further improvement of the glucose sensing performance. In particular, a high sensitivity of 1315 µA mM^−1^ cm^−2^ was measured for glucose determination using the amperometric method. This approach was also explored by Shu et al., who adopted a synergistic strategy by creating a binary metal–metal oxide system of Ni/NiO from an Ni-based MOF [84]. This material acted as a working electrode for the oxidation of glucose and resulted in a LOD of 0.8 µM. Wei and co-workers used CoCu-MOF as a precursor for generating CoCu oxide nanorods through the calcination process, which were embedded in copper foam (Figure 7a) [85]. The study revealed that glucose could be detected in the range 0.25–1200 µM (see Figure 7b,c). In addition, this sensor exhibited high sensitivity and excellent stability, as shown in Figure 7d–f. Moreover, this research group utilized the active material to detect glucose in blood and fruit juice, which gave reliable results. This synergic strategy represents a potential direction for the utilization of MOFs. Ding et al. created various architectures of binary metal oxides, such as CuO_x_@Co_3_O_4_, CuO_x_@NiO, CuO_x_@Fe_2_O_3_, CuO_x_@ZnO, and CuO_x_@CuO_x_, for glucose sensing [86]. Their results revealed that binary metal oxides have higher activities than those of single metal oxides. Furthermore, CuO_x_@Co_3_O_4_ showed the best performance among the investigated materials. Notably, this compound yielded a low LOD of 0.036 µM and a wide linear range of 0.1–1300.0 µM.

**Figure 8:**
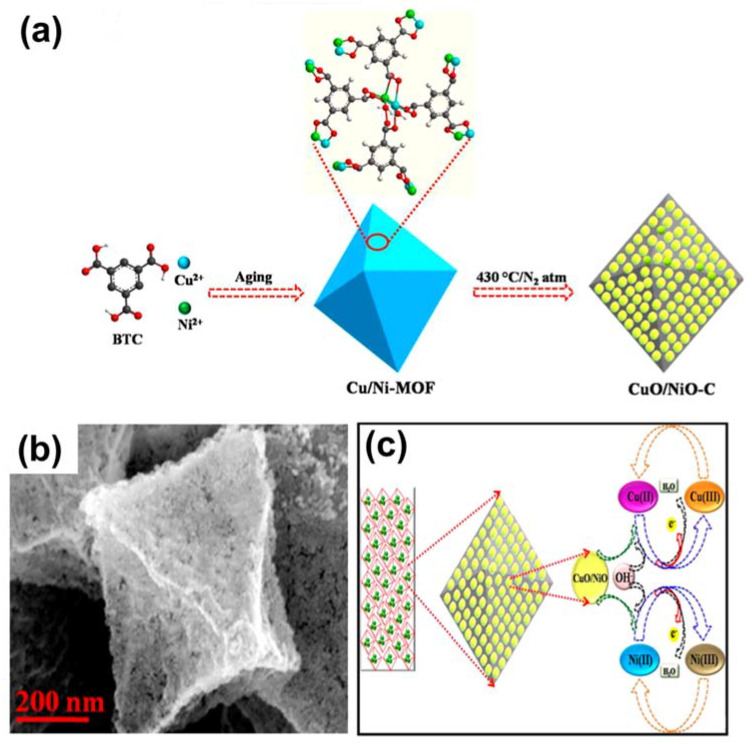
(**a**) Preparation of CuO/NiO-C, (**b**) SEM image of Cu/NiO-C, and (**c**) activation mechanism of CuO/NiO-C in alkaline media. Reproduced with permission from Ref. [87].

Another study related to MOF-derived binary metal oxides was presented by Archana and co-workers [87]. They utilized a NiCu-based MOF as a sacrificial agent to produce CuO/NiO-C, as shown in Figure 8a. The morphological structure of CuO/NiO-C is displayed in Figure 8b, which shows an octahedral shape. This strategy harnessed the synergic effect of binary oxides for glucose sensing. The research group also proposed the formation of active centers for glucose oxidation in the basic medium (Figure 8c); in particular, Ni^2+^ and Cu^2+^ were transformed into Ni^3+^ and Cu^3+^, respectively, which are the main active phases involved in glucose determination.

**Figure 9:**
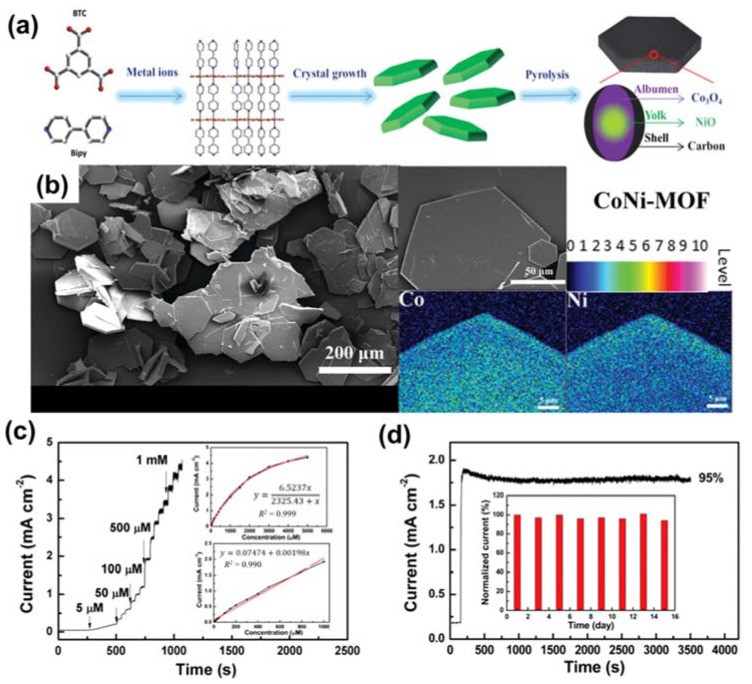
(**a**) Graphic representation of the preparation of YASNiCo@C. (**b**) SEM and elemental mapping images of CoNi‒MOF. (**c**) Current–time curve for the continuous addition of glucose to YASNiCo@C in alkaline media. (**d**) Amperometric plot for durability testing of YASNiCo@C. Reproduced with permission from Ref. [88].

Zhang et al. developed an interesting approach involving the use of a heterometallic MOF to create a binary metal oxide for glucose sensing [88]. The authors pyrolyzed a CoNi-MOF to generate a yolk–albumen–shell (YAS) architecture of NiCo@C (as presented in Figure 9a). The hexagonal shape of the CoNi-MOF can be observed in Figure 9b. The results revealed remarkable activity of the YASNiCo@C cathode with a low LOD of 0.75 M and a wide detection range of 5–1000 µM for glucose (as seen Figure 9c). Figure 9d shows the excellent durability of the electrode after 3500 *s* over eight cycles of use. This high performance was ascribed to the effect of bimetallic NiCo. In addition to Cu, Ni, Co-based MOFs, and Fe-MOFs are promising materials for efficient glucose determination. For instance, Abrori et al. used Fe-BDC as a sacrificial agent to prepare Fe_3_O_4_ for glucose identification using electrochemical methods [89]. This compound displayed a LOD of 15.57 M, high selectivity, and outstanding durability. Moreover, the authors proposed that the Fe(III) species in Fe_3_O_4_ are active centers for glucose oxidation. This study paved a new direction for the usage of Fe-based MOFs to fabricate anode materials for glucose determination.

**Figure 10:**
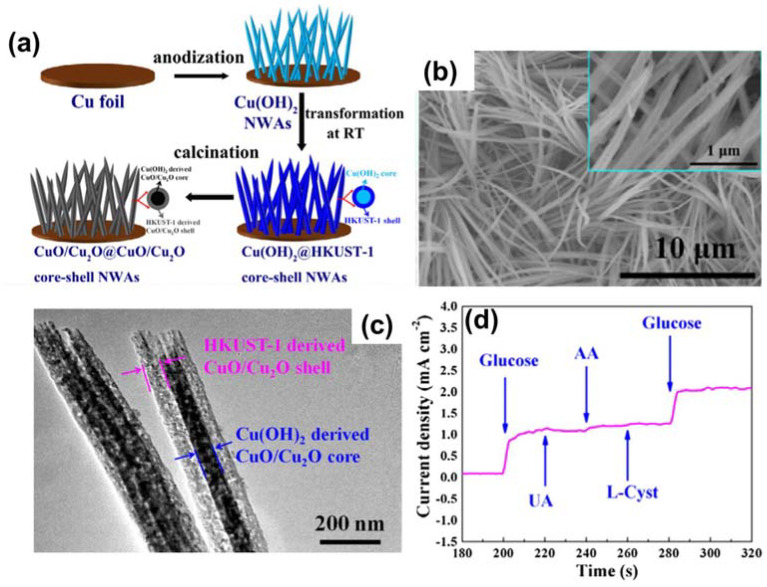
(**a**) Graphic representation of the preparation of CuO/Cu_2_O@CuO/Cu_2_O. (**b**) SEM and (**c**) TEM images of CuO/Cu_2_O@CuO/Cu_2_O. (**d**) Current–time response for continuous addition of 100 µM glucose to 5 µM UA, 5 µM AA, 5 µM L‒cystand, and 100 µM glucose using CuO/Cu_2_O@CuO/Cu_2_O in alkaline media. Reproduced with permission from Ref. [90].

Yu et al. reported an interesting architecture of CuO/Cu_2_O@CuO/Cu_2_O on a Cu template from a typical Cu-based MOF (HKUST-1), as presented in Figure 10a [90]. Notably, Cu(OH)_2_ NWs were first deposited on Cu foil, and then a Cu-MOF crystal was grown on the Cu(OH)_2_ NWs; this was followed by pyrolysis to generate the core–shell architecture (Figure 10b,c). This anode displayed interesting properties for glucose recognition, with a low LOD of 0.48 M and a sensitivity response of 10,090 mA mM^−1^ cm^−2^, which were determined from Figure 10d.

Other types of architectures based on MOFs have been reported for glucose detection. For example, Zhang and co-workers utilized CPO-27(Ni) as a sacrificial agent to prepare nickel phosphide, which was mixed with graphene (G) to form an efficient catalyst for glucose electro-oxidation [91]. Ni_2_P/G exhibited a high activity and low LOD of 0.44 µM, which was attributed to the beneficial effect of the Ni_2_P nanograins and graphene. Another study related to the usage of metal selenides as active materials for glucose sensing was reported by Jee et al. [92]. The authors deposited ZIF-67 on a Co substrate before carrying out oxidation and anion exchange to generate CoSe.

Finally, considering the high activity of MOF-related materials and their feasibility in glucose sensing applications, they can be mixed with other materials to further enhance their performance. Hence, based on the evidence from the literature, MOF-based materials are promising candidates for electrochemical and taste-sensing applications. The performances of MOF-based materials for taste sensors and glucose determination are summarized in Table 2.

## 4. MXenes for Glucose Detection

**Figure 11:**
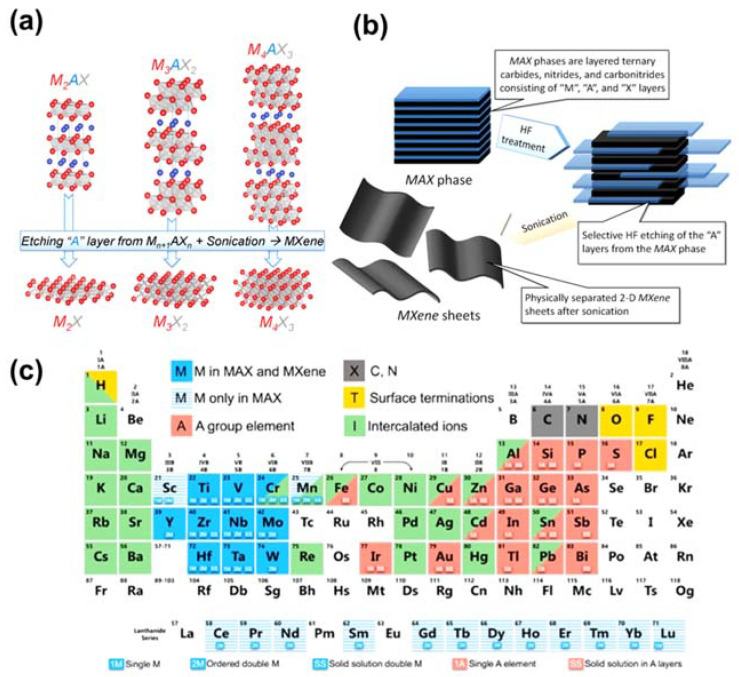
(**a**) Structural changes and formation of MAX and MXene phases using a fluorine-solution-based etching process with facile sonication. (**b**) Schematic illustration of the exfoliation process of MAX phases and MXenes involving HF treatment and sonication. (**c**) Periodic table highlighting the elements used to synthesize MAX phases and MXenes using both computational and experimental methods. Reproduced with permission from Ref. [94].

As state-of-the-art 2D materials, the transition metal carbide (TMC) and nitride family (TMN), also known as MXenes, have been obtained by selectively exfoliating the metallic bonds of MAX phases [93]. As shown in Figure 11a, to obtain MXenes from MAX phases, fluoride-containing acidic solutions of HF, LiF/HCl, NH_4_HF_2_, and KF were selected for etching the relatively strong M–A phase metal bonding and replacing it with weaker hydrogen bonds [94]. Thus, using fluorine-containing species in the etching process, the formula of the MXenes was changed to M_n+1_X_n_T_X_ because their surface became chemically terminated with oxygen (O), hydroxyl (–OH), and/or fluorine (–F) functional groups [95,96]. Furthermore, by modulating the layer distance through sequential exfoliation or delamination with organic solvents (e.g., tetramethylammonium hydroxide (TMAH), tetrabutylammonium hydroxide (TBAOH), and dimethyl sulfoxide (DMSO)) after the etching process, multilayer and monolayer MXenes were fabricated to enhance their sensitivity in sensing applications and store abundant lithium and sodium ions for battery applications (Figure 11b) [97]. As depicted in Figure 11c, hundreds of MAX phases have already been obtained using computational modeling or through experimentation [98]. However, following the graphene era, finding an equally promising prospective material for sensing applications remains a challenge. As an alternative to graphene, MXenes were chosen as appropriate sensing materials owing to their favorable properties, including hydrophilic surface, excellent conductivity, distinctive 2D structural characteristics, redox capability, superior strength, and remarkable flexibility [5,45]. This review briefly summarized various types of MXenes, including Ti_3_C_2_, which is perhaps the most famous member of the MXene family, and MXene-based composites for use in gas and glucose sensors employing electrochemical and optical methods.

### 4.1. MXenes

**Figure 12:**
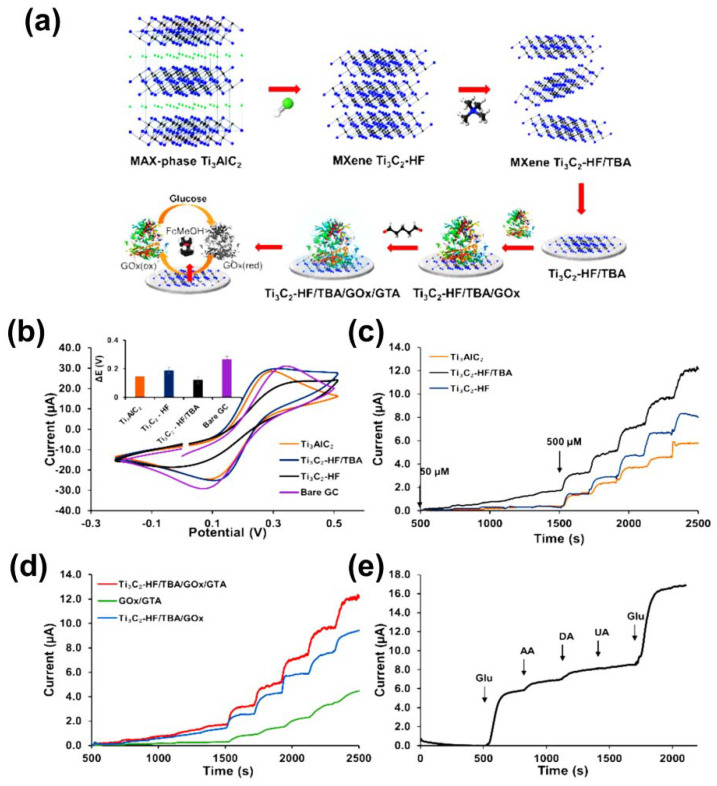
(**a**) Schematic illustration of synthetic process and glucose detection mechanism for Ti_3_C_2_‒HF/TBA/GO_x_/GTA. (**b**) Cyclic voltammetry (CV) curves for Ti_3_AlC_2_, Ti_3_C_2_‒HF, and Ti_3_C_2_‒HF/TBA compared with bare GC in 0.1 M KCl and 5 mM [Fe(CN)_6_]^4−/3−^ at a scan rate of 50 mV s^−1^; inset: peak‒to‒peak separation of Ti_3_AlC_2_, Ti_3_C_2_‒HF, and Ti_3_C_2_‒HF/TBA. Chronoamperometric curve for the addition of 50 µM of glucose every 200 s within the initial 1000 s, followed by addition of 500 µM of glucose every 200 s for the subsequent 1000 s at 0.15 V in PBS with 2 mM FcMeOH: (**c**) Ti_3_AlC_2_, Ti_3_C_2_‒HF, and Ti_3_C_2_‒HF/TBA; and (**d**) Ti_3_C_2_‒HF/TBA/GO_x_/GTA, GO_x_/GTA, and Ti_3_C_2_‒HF/TBA/GOx. (**e**) Chronoamperometry data obtained in the presence of various interfering species, including Glu, AA, DA, and UA using the Ti_3_C_2_‒HF/TBA glucose sensor with 2 mM FcMeOH at 0.15 V. Reproduced with permission from Ref. [110].

To prevent diabetes, which can develop into further complications, quantitative monitoring of glucose concentration in blood could help to diagnose critical disease or pre-symptom [99]. Owing to the development of 2D TMD and TMC materials, including molybdenum disulfide (MoS_2_), tungsten disulfide (WS_2_), and the MXene family, long-term stability, high sensitivity, low cost, and fast charge transfer of glucose sensors have been achieved, which are indispensable characteristics for diabetes diagnostics. These materials can accurately determine glucose concentration by virtue of their unique 2D structures, such as large surface areas, inert chemical substances, and abundant highly exposed edges [100,101,102,103]. Generally, glucose sensors are divided into two types: (1) optical and (2) electrochemical sensors [104,105,106]. As an intuitive method, the optical method can detect the reflected level of glucose by producing a change in the color of fluorescent dye [107]. The electrochemical method has been explored to facilitate the transfer of electrons and electrical contact on the surface [108]. However, poor electrical performance is related to the response time, limiting the capability of the electrochemical glucose sensor [109]. As a means to improve the electrical properties, we subjected Ti_3_C_2_ to a sequential delamination process using organic solvents, especially TBAOH, after selective HF etching. This process yielded a promising electrochemical glucose sensor with excellent electrical performance, as verified using various electrochemical measurements introduced by Hui et al. [110]. The overall preparation process is shown in Figure 12a, in which Ti_3_AlC_2_ was immersed in 40% HF with vigorous stirring for 7 days. Following HF treatment, the morphology of the as-prepared Ti_3_C_2_ was altered to form a stacked, accordion-like structure. The as-prepared Ti_3_C_2_ was then continuously stirred with 50 mL of TBAOH for 1 h. Finally, Ti_3_C_2_-HF/TBA was obtained after 30 min of ultrasonication followed by drying for 24 h in a vacuum oven. In order to fabricate single-or few-layer MXenes, TBA^+^, the large cation comprising TBAOH, was inserted into the interlayer of MXenes to expand the space between layers. For electrochemical measurements, Ti_3_AlC_2_ was immersed in dimethylformamide (DMF) and sonicated for 1 min to obtain a homogeneous mixture; 0.1 mg of glucose oxidase (GOx) was added to prepare the final materials (Ti_3_AlC_2_/GO_x_/GTA (GTA: glutaraldehyde), Ti_3_C_2_-HF/GO_x_/GTA, and Ti_3_C_2_-HF/TBA/GO_x_/GTA), which were then deposited on GCEs. From the cyclic voltammograms shown in Figure 12b, Ti_3_C_2_-HF/TBA exhibited lower peak-to-peak separation (∆E_p_) than that of Ti_3_AlC_2_, Ti_3_C_2_-HF, and bare GC electrode, which is associated with faster heterogeneous electron-transfer (HET) capability.

Chronoamperometric measurements are widely used for evaluating glucose detection response and determining sensitivity differences. Upon consecutively adding glucose, Ti_3_C_2_-HF/TBA exhibited an outstanding glucose detection response compared with that of the other examined materials because of its single- and few-layer composition introduced by delamination using TBAOH. The advantages for detecting glucose response, such as higher and more active surface area, short interaction distances, and superior electron transfer, enhance the electrical conductivity between the enzyme and the electrode. Based on the chronoamperometry measurement with sequential glucose addition, compared with GOx/GTA, Ti_3_C_2_-HF/TBA/GOx/GTA exhibited remarkable electrochemical properties by incorporating an MXene on a GC electrode (Figure 12c,d). As shown Figure 12e, when determining the selectivity of biosensor, interfering species such as L-ascorbic acid, dopamine hydrochloride, and uric acid did not lead to remarkable current changes, while the biosensor clearly preferred to react with glucose rather than the interfering species in chronoamperometry measurements at 0.15 V.

**Figure 13:**
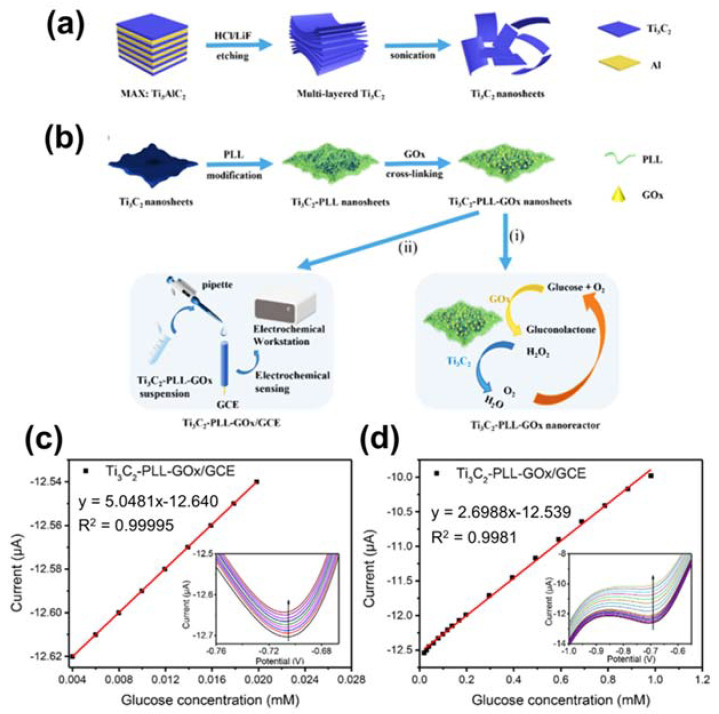
Graphic representation of the preparation of Ti_3_C_2_‒PLL‒GOx nanoreactor. (**a**) The Ti_3_C_2_ MXene nanolayers were gained after etching of the Al layer from Ti_3_AlC_2_. (**b**) The modification of T_3_C_2_‒PLL‒GOx nanolayers by PLL and GOx for cascade glucose oxidation (i) and electrochemical glucose sensing (ii). Linear correction plots between current and glucose concentration obtained from the Ti_3_C_2_‒PLL‒GOx/GCE sensors with glucose concentration in a range of (**c**) 4.0‒20 µM and (**d**) 0.02–1.1 mM. CV responses of glucose at various concentrations are inserted in plots. Reproduced with permission from Ref. [106].

Recently, Wu and co-workers created Ti_3_C_2_ thin films from Ti_3_AlC_2_, followed by functionalizing by poly-L-lysine (PLL) and glucose oxidise (GOx) for glucose determination, as seen in Figure 13a,b [106]. Ti_3_C_2_-PLL-GOx was coated on GCE and acted as an anode for glucose oxidation. Electrode activity was investigated by CV methods with the successive injection of glucose with various concentrations. The outcome revealed that glucose concentration was accelerated, the reduction peak current decelerated (Figure 13c,d). This is because glucose consumes O_2_ to decompose then enzymes, leading to the decelerating of O_2_ reduction current.

### 4.2. MXene-Based Composites

**Figure 14:**
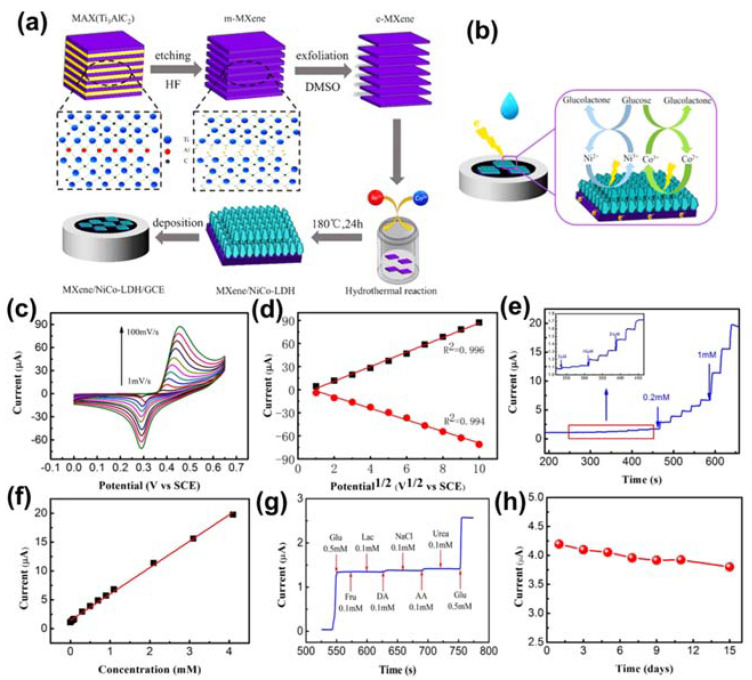
(**a**) Preparation process of MXene/NiCo‒LDH/GCE. (**b**) Mechanism of MXene/NiCo‒LDH‒GCE electrode reacting with glucose in alkaline solution. (**c**) Cyclic voltammetry curves of MXene/NiCo‒LDH/GCE in 0.1 M KOH at different scan rates (from 1–100 mV/s). (**d**) Near-linear relationship of the anodic peak current (I_a_) and cathodic peak current (I_c_) vs. the square root of the scan rate. (**e**) Chronoamperometry curve of MXene/NiCo‒LDH/GCE (glucose concentration of 2 µM to 1 mM) in 0.1 M KOH at 0.45 V, and current response for the low concentration range of glucose (inset). (**f**) Calibration plot of the response vs. glucose concentration for MXene/NiCo‒LDH/GCE. (**g**) Chronoamperometry data for the MXene/NiCo‒LDH/GCE glucose sensor in the presence of various interfering species, including Glu, Fru, Lac, DA, NaCl, AA, and urea, in 0.1 M KOH solution at 0.45 V. (**h**) Current–time response of MXene/NiCo‒LDH/GCE toward 1 mM glucose in 0.1 M KOH solution over 15 days. Reproduced with permission from Ref. [115].

Through fabricating MXene-based composites, super-sensitive glucose sensors with improved mechanical, electrical, and optical performance have been realized [111,112,113]. For example, Zhu et al. fabricated Ti_3_C_2_ nanosheets bounded to red-emitting carbon dots in order to enhance the selectivity and achieve fast response times of an optical glucose sensor [114]. In contrast with the intuitive optical method, this method provides a straightforward means to perceive low levels of blood glucose during enzymatic reactions. To counteract the drawbacks of the optical method, the electrochemical method holds substantial potential in terms of scalability. Therefore, the authors introduced Ni/Co layered double hydroxide (NiCo-LDH) nanosheets on a conductive MXene by using a simple hydrothermal method to produce a promising electrochemical glucose sensor [115]. The entire fabrication process and reaction mechanism between the MXene/NiCo-LDH composite and glucose is shown in Figure 14a,b. To prepare the MXene/NiCo-LDH composite, Ti_3_AlC_2_ was first immersed in HF at room temperature to synthesize multilayered MXene (m-MXene), and then, by mixing with DMSO, single-layer MXene (e-MXene) was obtained. The as-prepared e-MXene, nickel and cobalt precursors, and CTAB were simultaneously combined in a mixed solvent under hydrothermal processing at 180 °C for 24 h to obtain the composite. For the electrochemical experiments, the MXene/NiCo-LDH was deposited onto a GCE. As shown in Figure 14c, the electrochemical mechanism of MXene/NiCo-LDH was validated through CV at different scan rates (from 1–100 mV/s) in 0.1 M KOH solution. Additionally, the linear relationships for the anodic peak current (I_a_) and cathodic peak current (I_c_) are shown in Figure 14d. The chronoamperometric curve obtained upon electrochemical testing every 25 s of gradually increasing the concentration of glucose in the alkaline solution from 2 µM to 1 Mm, along with the calibration curve for current response from 0.002–4.096 mM, are shown in Figure 14e,f. The MXene/NiCo-LDH sensor exhibited significantly improved electrochemical properties by reducing the response time (3 s) such that the current reached a steady state. To verify the selectivity, a critical parameter of the glucose sensor, various inorganic and organic substances including NaCl, lactose, ascorbic acid, fructose, dopamine, and urea were combined with glucose in blood and subjected to chronoamperometry in 0.1 M KOH solution. The amperometric response of MXene/NiCo-LDH/GCE for 0.5 mM glucose and 0.1 mM of the respective interfering species demonstrated its excellent selectivity for glucose (Figure 14g). Moreover, the MXene/NiCo-LDH/GCE glucose sensor exhibited excellent selectivity and long-term stability, as demonstrated through its current response, which fell to only 92.5% of its initial value after 7 days and retained 92.5% of its initial value after 15 days (Figure 14h).

Another study was recently reported by Bi and co-workers [116]. The authors prepared a coral-like architecture of Au NPs on the surface of MXene, which is favorable for exposed active centers. This material was deposited on GCE with the support of Nafion solution. Au/MXene/Nafion/GCE operates as a working electrode for glucose determination. The performance of Au/MXene/Nafion/GCE was evaluated via amperometric measurements with continuous injection of glucose solution. The outcome indicated that this electrode has a low LOD of 0.2 mM with a 1–12 mM linear range. In addition, this material exhibited the repeatable property for glucose detection with five samples, which were fabricated by the same process.

**Figure 15:**
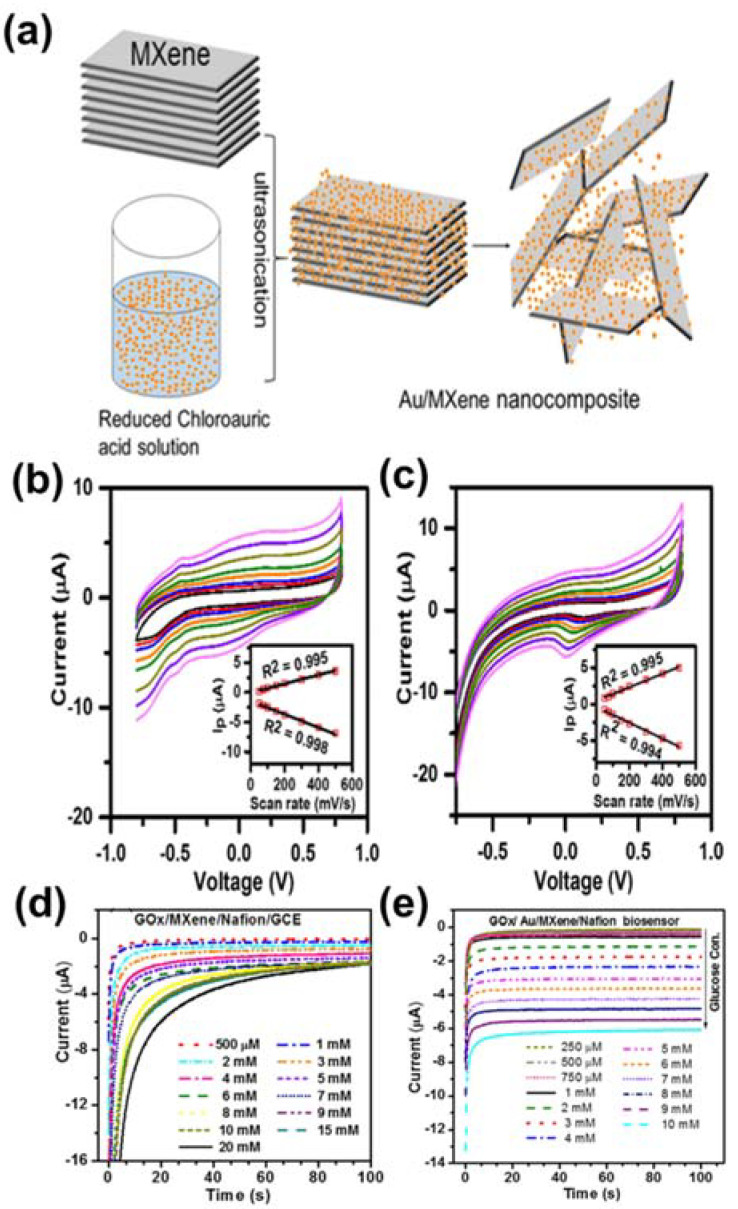
(**a**) Schematic preparation to design Au/MXene nanocomposite. Cyclic voltammetry (CV) curves of (**b**) GOx/Au/MXene/Nafion/GCE and (**c**) GOx/MXene/Nafion/GCE biosensors at different scan rates (from 50–500 mV/s) and corresponding near‒linear relationship of the redox peak current (I_p_) vs. the square root of the scan rate (inset). Chronoamperometry curves for addition of different concentrations of glucose of (**d**) GOx/MXene/Nafion/GCE and (**e**) GOx/Au/MXene/Nafion/GCE electrodes at −0.402 V. Reproduced with permission from Ref. [112].

To increase the sensitivity, stability, repeatability and reproducibility of glucose sensor, Rakhi et al. innovatively fabricated the immobilization of GOx enzyme on Nafion solubilized Au nanoparticles and MXene nanosheet composite over GCE, which is called GOx/Au/MXene/Nafion/GCE glucose sensor [112]. Figure 15a presented a schematic illustration synthesis of Au/MXene composite by a mere drop-casting process. The finding revealed that the fabricated GOx/Au/MXene/Nafion/GCE electrode shows relatively faster electron transfer than GOx/MXene/Nafion/GCE electrode, whether Au nanoparticles that tend to shift redox peak toward to lower potential for facilitating the electron activity anchored on the surface of MXene is existed or not (Figure 15b,c). Furthermore, as shown in Figure 15d,e, from chronoamperometry curves of GO_X_/Au/MXene/Nafion/GCE glucose sensor, it can reach a stable state for current within 10s, while GO_X_/MXene/Nafion/GCE glucose sensor can stabilize the current within 20 s for detecting added different concentration of glucose at a constant voltage of −0.402 V. As a result, GO_X_/MXene/Nafion/GCE glucose sensor definitively improved the properties, following the excellent sensitivity of 4.2 µM mM^−1^ cm^−2^ as well as lower detection limit 5.9 µM extracted from diverse electrochemical analysis. The performances of MXenes-based materials for glucose detection are collected in Table 3.

## 5. Conclusions and Future Outlook

In this review, we outlined the outstanding properties of MOFs and MXenes materials, which have emerged as powerful templates for chemical and biological sensors. We presented interesting studies that incorporated MOFs into taste sensors and also described the structures of MXenes as well as the synthetic techniques used to prepare them. We provided ample evidence to demonstrate the ability of MXenes to act as electrochemical sensors for glucose determination. Moreover, the operating principle of taste sensors and their mechanisms of glucose detection were discussed. Owing to their unique properties, MOFs and MXenes show great potential for application in electronic tongues. The growth of uniform MOF crystals on various templates (Au, Ni, Cu, …) with a tunable pore size can create a sensor array system with high selectivity to different biomolecules. While MXenes with tunable surface characteristics could easily be deposited on the substrates to prepare a flexible sensors array for detecting organic compounds. In addition, MXenes could accelerate the performance of the electrochemical sensor of MOFs due to their excellent electrical conductivity. These mentioned evidence exhibit the possibility of a sensor array system using MOFs and MXenes towards simultaneous detection of multiple tastes. However, the low selectivity of MXenes based sensors is still a significant challenge, while the strong attachment of MOFs on templates is a challenging task. In addition, the synthetic method structure‒property relationship of MXenes and MOFs that governs the performance of the sensors should be systematically investigated. Furthermore, various strategies must be developed to improve the sensitivity, selectivity, and durability of the MOF- and MXene-based electrochemical sensors.

## Figures and Tables

**Table 1:** Analytes and properties of five tastes.

Taste	Analytes	Properties
Saltiness	KCl, NaCl, metal ions	Providing of mineral
Sweetness	Glucose, sucrose, saccharin	Source of energy
Sourness	Acids: HCl, CH_3_CHOOH, etc.	Activation of metabolism
Bitterness	Caffein, quinine, etc.	Notice of toxicity
Umami	Monosodium glutamate	Providing of indispensable amino acids

**Table 2:** Summary works MOF-based tastes sensors and glucose detection.

Samples	Analytes	Method	LOD	Ref
MOF-76 [In(OH)(bdc)n	Sucrose, caffeine, citric acid, NaCl, monosodium glutamate	PL emission spectra	Not given	[55]
(+)-PAN-chelated [In(OH)(bdc)]	Sucrose, caffeine, citric acid, NaCl, monosodium glutamate	PL emission spectra	Not given	[56]
CPO-27(Ni)	Glucose	Electrochemical techniques	1.46 µM	[26]
Ni-MIL-77	Glucose	Electrochemical techniques	0.25 µM	[27]
Conductive Ni-MOF	Glucose	Electrochemical techniques	0.66 µM	[61]
AgNPs/MOF-74(Ni)	Glucose	Electrochemical techniques	4.7 µM	[62]
Au/BTC	Glucose	Electrochemical techniques	1.5 µM	[63]
Ni-MOF/CNTs	Glucose	Electrochemical techniques	0.82 µM	[4]
ZIF-67 HNPs	Glucose	Electrochemical techniques	0.96 µM	[64]
Ag@ZIF-67	Glucose	Electrochemical techniques	0.66 µM	[65]
Co-BDC-3Gr	Glucose	Electrochemical techniques	5.39 µM	[66]
Co-MOF/CC	Glucose	Electrochemical techniques	150 µM	[67]
NiCo-MOF/CC	Glucose	Electrochemical techniques	100 nM	[68]
Ni/Co-TCPP	Glucose	Electrochemical techniques	0.3 µM	[69]
Co-MOF/NF	Glucose	Electrochemical techniques	1.3 nM	[70]
NiCo-MOF nanoflake	Glucose	Electrochemical techniques	0.29 nM	[71]
Co/Ni-MOF	Glucose	Electrochemical techniques	0.047 µM	[72]
Cu-MOF/CPE	Glucose	Electrochemical techniques	0.11 µM	[73]
Cu-MOF	Glucose	Electrochemical techniques	0.01 µM	[74]
Cu-MOF/EG	Glucose	Electrochemical techniques	0.58 µM	[75]
Cu-MOF/CNHs	Glucose	Electrochemical techniques	0.078 µM	[76]
Cu-MOF/CNT	Glucose	Electrochemical techniques	0.4 µM	[77]
2D-Fe-BTC	Glucose	Electrochemical techniques	0.039 µM	[78]
Fe-MIL-88NH_2_	Glucose	Electrochemical techniques	0.48 M	[79]
MIL-53(Fe)	Glucose	Electrochemical techniques	0.039 µM	[80]
Ni@C	Glucose	Electrochemical techniques	50 nM	[81]
CuO	Glucose	Electrochemical techniques	0.15 µM	[82]
Co_3_O_4_/rGO	Glucose	Electrochemical techniques	0.4 µM	[83]
Ni/NiO	Glucose	Electrochemical techniques	0.8 µM	[84]
CoCu oxide nanorods	Glucose	Electrochemical techniques	0.72 μM	[85]
CuO_x_@Co_3_O_4_	Glucose	Electrochemical techniques	0.036 µM	[86]
CuO/NiO-C	Glucose	Electrochemical techniques	37 nM	[87]
YASNiCo@C	Glucose	Electrochemical techniques	0.75 M	[88]
Fe_3_O_4_	Glucose	Electrochemical techniques	15.57 M	[89]
Ni_2_P/G	Glucose	Electrochemical techniques	0.44 μM	[90]
CoSe/ZIF-67	Glucose	Electrochemical techniques	1.53 µM	[91]
CuO/Cu_2_O@CuO/Cu_2_O	Glucose	Electrochemical techniques	0.48 M	[92]

**Table 3:** MXenes based electrochemical sensors for glucose detection.

Samples	Analytes	Methods	LOD	Ref
Ti_3_C_2_-HF/TBA/GOx/GTA	Glucose	Electrochemical technique	23.0 µM	[110]
Ti_3_C_2_-PLL-GOx	Glucose	Electrochemical technique	2.6 µM	[106]
MXene/NiCo-LDH	Glucose	Electrochemical technique	0.53 µM	[115]
Au/MXene/Nafion/GCE	Glucose	Electrochemical technique	0.2 mM	[116]
GOx/Au/MXene/Nafion/GCE	Glucose	Electrochemical technique	5.9 µM	[112]

## Data Availability

Not applicable.
